# Diagnostic Accuracy of Oral Scrape Cytology for Detection of Premalignant and Malignant Oral Lesions: A Cross-Sectional Comparative Study

**DOI:** 10.7759/cureus.110060

**Published:** 2026-06-01

**Authors:** Sonia Oraon, Deepali Tirkey, Arvind Kumar, Sunil Kumar Mahto

**Affiliations:** 1 Pathology, Rajendra Institute of Medical Sciences, Ranchi, IND

**Keywords:** diagnostic accuracy, histopathology, oral cancer, oral scrape cytology, premalignant lesions, tobacco exposure

## Abstract

Background: Oral scrape cytology has emerged as a simple, cost-effective, and minimally invasive diagnostic tool for the evaluation of oral mucosal lesions. The present study was undertaken to evaluate the diagnostic efficacy of oral scrape cytology in detecting premalignant and malignant oral cavity lesions by correlating cytological findings with histopathological diagnoses.

Methods: Our study was a hospital-based cross-sectional study conducted in the Departments of Pathology and Dentistry at Rajendra Institute of Medical Sciences (RIMS), Ranchi, Jharkhand, from July 2024 to January 2026. A total of 129 patients with clinically suspicious oral cavity lesions underwent oral scrape cytology followed by histopathological evaluation. Sensitivity, specificity, positive predictive value (PPV), negative predictive value (NPV), and diagnostic accuracy were calculated using histopathology as the reference standard.

Results: The mean age of the study population was 51.47±15.94 years, with a male predominance and a high prevalence of tobacco exposure. Oral scrape cytology demonstrated a sensitivity of 80.4%, a specificity of 80.7%, a PPV of 69.8%, a NPV of 88.2%, and an overall diagnostic accuracy of 80.6%. There was a statistically significant correlation between cytological and histopathological diagnoses.

Conclusion: Our findings demonstrate that oral scrape cytology is a reliable, minimally invasive, and cost-effective adjunctive diagnostic modality for the evaluation of clinically suspicious oral lesions. Although histopathological examination remains the gold standard for definitive diagnosis, oral scrape cytology may serve as an effective screening and triage tool, especially in resource-constrained settings and high-risk populations with significant tobacco exposure.

## Introduction

Oral cancer is a significant global health burden and one of the most common cancers of the head and neck. Based on the Global Cancer Observatory (GLOBOCAN) 2020 estimates, cancers of the lip and oral cavity were responsible for about 377,000 new cases and around 177,000 deaths globally, with one of the highest incidence rates in South-Central Asia [1]. Oral cancer is a public health problem in India and accounts for a significant proportion of cancer-related morbidity and mortality because of the extensive use of smoked (cigarettes, beedis) and smokeless tobacco, areca nut, betel quid chewing, and socioeconomic factors [2]. Oral squamous cell carcinoma (OSCC) accounts for over 90% of oral cancers and mostly arises through a multistep process characterized by epithelial dysplasia, accumulation of genetic changes, and modification of the tumor microenvironment, leading to invasion [3]. Oral potentially malignant disorders (OPMDs), such as leukoplakia, erythroplakia, and oral lichen planus (OLP), are accepted precursors with varying risks of malignant transformation [4]. Early recognition of high-risk neoplasia and superficial malignancies has significant prognostic implications, favoring better outcomes.

While histopathological examination is considered the gold standard diagnostic tool, biopsy is an invasive procedure that requires trained personnel and may not be available in resource-limited settings or for patients who are unwilling to undergo invasive procedures [5]. This has led to increased interest in minimally invasive adjunctive diagnostic procedures that can aid in the early diagnosis and triage of suspicious oral lesions. Oral scrape cytology involves the simple collection of exfoliated epithelial cells from oral mucosal lesions and is a rapid, microscopic evaluation that is easy, quick, and inexpensive. Oral cytology has been shown to be a promising diagnostic tool for the detection of oral epithelial dysplasia and OSCC in several studies [6]. Rates of false-negative results have been reported between 60% and 95%, and this high variability depends on sampling technique, lesion characteristics, and the criteria used for cytological interpretation [7,8]. False-negative results are seen particularly when well-differentiated keratinizing lesions are sampled and when only a few atypical basal cells are exfoliated. Although there is a positive trend, information on the diagnostic value of oral scrape cytology from tertiary care centers serving high-risk Indian populations remains limited. The technique should be evaluated at the institutional level because patterns of tobacco exposure, the spectrum of lesions, and healthcare accessibility vary across settings. Therefore, our study was designed to evaluate the sensitivity, specificity, predictive values, and overall usefulness of oral scrape cytology in the management of premalignant and malignant oral lesions as an adjunctive screening and diagnostic tool.

## Materials and methods

Our study was a hospital-based cross-sectional study conducted in the Departments of Pathology and Dentistry at Rajendra Institute of Medical Sciences (RIMS), Ranchi, Jharkhand, from July 2024 to January 2026. Patients with clinically suspicious oral cavity lesions (white patches, red patches, ulcerative lesions, non-healing ulcers, and exophytic growths) were evaluated. This was an all-comer approach with consecutive sampling, meaning that all patients who met the inclusion criteria during the study period were recruited after providing written informed consent. Ethical clearance was obtained from the Institutional Ethics Committee of RIMS, Ranchi. Patients who underwent oral scrape cytology followed by histopathological evaluation were included in the analysis. Patients who had received previous radiotherapy or chemotherapy, previous oral cancer treatment, or had a malignancy elsewhere were excluded. Sample size estimation was performed using the formula N=4p(1-p)/d², where p represents the reported sensitivity of oral scrape cytology (p=0.8 [9]), and d represents the allowable margin of error. A margin of error of 8% was considered for the study. Based on the sensitivity reported by Babshet et al. [9] for the detection of premalignant and malignant oral lesions, the minimum required sample size was calculated to be 100. An additional 10% was added to compensate for dropouts. Accordingly, a total of 129 samples were ultimately included in the final analysis.

For cytological evaluation, patients were asked to thoroughly rinse the oral cavity before sampling. All representative areas of the lesions were scraped with a wooden spatula using uniform pressure until pinpoint bleeding was noted, thereby obtaining transepithelial samples. Smears were prepared on clean, grease-free slides. The smears were either wet-fixed and stained with the Papanicolaou stain or air-dried and stained with Leishman and Giemsa stains. Cytological evaluation included assessment of hyperchromasia, chromatin pattern, nuclear pleomorphism, nuclear-cytoplasmic ratio, abnormal mitotic activity, and cellularity.

Cytological results were then correlated with histopathological diagnoses, which served as the reference standard. Biopsy specimens were processed using standard paraffin-embedding techniques, stained with hematoxylin and eosin, and examined histopathologically. Data were analyzed using MedCalc software (MedCalc Software Ltd., Ostend, Belgium). Sensitivity, specificity, positive predictive value (PPV), negative predictive value (NPV), and diagnostic accuracy were determined. When applicable, data were analyzed using the chi-square test or Fisher’s exact test, and a p-value <0.05 was considered statistically significant. Institutional Ethics Committee approval was obtained on February 1, 2025 (IEC Reference No. 77), and all participants provided written informed consent before participation in the study.

## Results

We included 129 patients with clinically suspicious oral cavity lesions in our study. The mean age of the study population was 51.47±15.94 years, and the majority of patients belonged to the 41-60-year age group, accounting for 46 (35.7%) cases. We observed a marked male predominance, with 92 (71.3%) males and 37 (28.7%) females, yielding a male-to-female ratio of approximately 2.5:1. Tobacco exposure was highly prevalent, particularly chewing tobacco in 51 (39.5%) patients, followed by smoking in 38 (29.5%) and combined tobacco use in 25 (19.4%) patients. Alcohol consumption was reported in 53 (41.1%) cases (Table 1).

**Table 1:** Demographic characteristics and risk factor profile of study participants (n=129)

Variable	Category	n (%)
Age group	21-40 years	39 (30.2%)
	41-60 years	46 (35.7%)
	61-80 years	44 (34.1%)
Sex	Male	92 (71.3%)
	Female	37 (28.7%)
Tobacco use	Chewing	51 (39.5%)
	Smoking	38 (29.5%)
	Both	25 (19.4%)
	None	15 (11.6%)
Alcohol use	Yes	53 (41.1%)
	No	76 (58.9%)

We observed that the hard palate was the most frequently involved anatomical site, seen in 33 (25.6%) patients, followed by the buccal mucosa in 28 (21.7%) and the tongue in 26 (20.2%) cases. The most common clinical presentations were red patches and ulcerative lesions, each observed in 34 (26.4%) patients, followed by white patches in 32 (24.8%) and exophytic growths in 29 (22.5%) cases (Table 2).

**Table 2:** Clinical characteristics and anatomical distribution of oral lesions

Variable	Category	n (%)
Anatomical site	Hard palate	33 (25.6%)
	Buccal mucosa	28 (21.7%)
	Tongue	26 (20.2%)
	Floor of mouth	21 (16.3%)
	Gingiva	21 (16.3%)
Clinical presentation	Red patch	34 (26.4%)
	Ulcerative lesion	34 (26.4%)
	White patch	32 (24.8%)
	Growth	29 (22.5%)

In our study, oral scrape cytology identified malignant lesions in 53 (41.1%) cases and premalignant lesions in 47 (36.4%) cases. Histopathological examination confirmed malignant lesions in 46 (35.7%) patients and premalignant lesions in 55 (42.6%) patients. We observed that moderately differentiated squamous cell carcinoma was the most common histopathological subtype (Table 3).

**Table 3:** Distribution of cytologic and histopathologic diagnoses

Diagnostic category	Cytology, n (%)	Histopathology, n (%)
Negative/benign	29 (22.5%)	28 (21.7%)
Premalignant lesions	47 (36.4%)	55 (42.6%)
Malignant lesions	53 (41.1%)	46 (35.7%)

The present analysis demonstrated 37 true-positive and 67 true-negative cases on cytology-histopathology correlation, while 16 cases were false-positive and nine cases were false-negative. Discordant findings were predominantly associated with premalignant lesions and well-differentiated squamous cell carcinoma. We observed a statistically significant association between cytological and histopathological diagnoses (χ²=38.7, p<0.001) (Table 4).

**Table 4:** Correlation between oral scrape cytology and histopathological diagnosis χ²(1)=38.7, p<0.001

Oral scrape cytology	Histopathology malignant, n (%)	Histopathology non-malignant, n (%)	Total, n (%)
Malignant	37 (28.7%)	16 (12.4%)	53 (41.1%)
Non-malignant	9 (7.0%)	67 (51.9%)	76 (58.9%)
Total	46 (35.7%)	83 (64.3%)	129 (100%)

Our findings showed a sensitivity of 80.4%, specificity of 80.7%, PPV of 69.8%, and NPV of 88.2%, with an overall diagnostic accuracy of 80.6%. We observed that oral scrape cytology serves as a useful and minimally invasive screening adjunct in the evaluation of premalignant and malignant oral lesions (Table 5).

**Table 5:** Diagnostic performance and concordance analysis of oral scrape cytology

Parameter	Value (95% CI)
Sensitivity	80.4% (66.8%-89.3%)
Specificity	80.7% (71.0%-87.8%)
Positive predictive value	69.8% (56.5%-80.5%)
Negative predictive value	88.2% (79.0%-93.6%)
Diagnostic accuracy	80.6% (73.0%-86.5%)
Concordant cases	104 (80.6%)
Discordant cases	25 (19.4%)

## Discussion

Early identification of oral premalignant and malignant lesions remains essential for reducing the morbidity and mortality associated with oral cancer, particularly among high-risk populations with chronic tobacco exposure. In our study, oral scrape cytology demonstrated an overall diagnostic accuracy of 80.6%, with sensitivity and specificity of 80.4% and 80.7%, respectively. We observed a PPV of 69.8% and an NPV of 88.2%, supporting the utility of oral scrape cytology as a screening and triage modality for clinically suspicious oral lesions, especially in resource-constrained settings where immediate biopsy facilities may be limited or unavailable. The majority of patients in our study belonged to the fifth and sixth decades of life, with 92 (71.3%) men. Similar demographic findings were reported by Besra et al. and Mehrotra et al., who also observed a higher prevalence of oral premalignant and malignant lesions among middle-aged men with chronic tobacco exposure [10,11]. In our cohort, tobacco use, particularly smokeless tobacco consumption, was highly prevalent and reflected the established etiological role of tobacco in oral carcinogenesis. Warnakulasuriya et al. also emphasized the strong association between tobacco-related habits and the development of OPMDs and OSCC [4].

We observed that the hard palate and buccal mucosa were the most frequently involved anatomical sites. Comparable findings were reported by Naveen Kumar et al. in patients with tobacco-associated oral lesions [12]. Clinically, red patches and ulcerative lesions each accounted for 34 (26.4%) cases, reflecting their increased dysplastic and malignant potential. Previous studies have similarly identified erythroplakia and ulceroproliferative lesions as high-risk clinical entities requiring prompt evaluation [13]. Histopathological correlation in our study demonstrated 37 (28.7%) true-positive and 67 (51.9%) true-negative cases, whereas false-positive and false-negative results were observed in 16 (12.4%) and nine (7.0%) cases, respectively. Concordance between cytological and histopathological findings was highest in overtly malignant lesions exhibiting marked nuclear atypia. Discordant findings were predominantly associated with premalignant lesions and well-differentiated keratinizing squamous cell carcinomas. Cytological limitations contributing to discordance included hyperkeratinization, low cellular yield, superficial sampling, overlapping inflammatory cells, and obscuring necrotic debris, all of which hinder adequate visualization of atypical basal and parabasal cells. Well-differentiated keratinizing lesions often exfoliate predominantly superficial mature squamous cells with minimal atypia, thereby increasing the likelihood of false-negative interpretation. Similar observations were reported by Sciubba et al., who noted that hyperkeratinized lesions frequently yield inadequate transepithelial sampling and reduce diagnostic sensitivity in oral scrape cytology [6]. Recognition of these cytomorphological limitations is important to avoid underdiagnosis and to ensure that clinically suspicious lesions undergo histopathological confirmation despite negative or equivocal cytology findings. Babshet et al. also observed that reactive inflammatory atypia may occasionally mimic dysplastic changes and contribute to false-positive cytological diagnoses [9].

The sensitivity observed in our study was comparable to the 79.2% sensitivity reported by Idrees et al. using liquid-based oral brush cytology [8]. However, higher sensitivity values ranging from 90% to 95% have been documented by Besra et al. and Maraki et al. using brush biopsy techniques with improved sampling methods [10,14]. Our specificity of 80.7% was lower than the specificity exceeding 90% reported by Navone et al. in oral brush biopsy studies [15]. Nevertheless, the diagnostic indices observed in our cohort remain clinically acceptable and support the role of oral scrape cytology as an adjunctive diagnostic modality.

Moderately differentiated squamous cell carcinoma represented the predominant malignant subtype in our study, consistent with findings from other Indian tertiary-care analyses [16]. The minimally invasive nature, rapid turnaround time, and low procedural cost of oral scrape cytology make it particularly valuable in community screening programs and resource-constrained healthcare settings. However, the single-center design, relatively limited sample size, and absence of adjunctive molecular techniques may have influenced diagnostic accuracy and contributed to discordant findings.

## Conclusions

Our findings demonstrate that oral scrape cytology is a reliable, minimally invasive, and cost-effective adjunctive diagnostic modality for the evaluation of clinically suspicious oral lesions. The technique showed good sensitivity, specificity, and overall diagnostic accuracy, with a particularly high NPV for the detection of premalignant and malignant lesions. Although histopathological examination remains the gold standard for definitive diagnosis, oral scrape cytology may serve as an effective screening and triage tool, especially in resource-constrained settings and high-risk populations with significant tobacco exposure.
